# Coumarins inhibit η-class carbonic anhydrase from *Plasmodium falciparum*

**DOI:** 10.1080/14756366.2022.2036986

**Published:** 2022-02-09

**Authors:** Simone Giovannuzzi, Viviana De Luca, Alessio Nocentini, Clemente Capasso, Claudiu T. Supuran

**Affiliations:** a Neurofarba Department, Pharmaceutical and Nutraceutical Section, University of Florence, Sesto, Italy; b Department of Biology, Agriculture and Food Sciences, CNR, Institute of Biosciences and Bioresources, Napoli, Italy

**Keywords:** Carbonic anhydrase, inhibitor, coumarins, *Plasmodium falciparum*, anti-protozoal agents

## Abstract

Coumarins were discovered to act as inhibitors of α-carbonic anhydrases (CAs, EC 4.2.1.1) after undergoing hydrolysis mediated by the esterase activity of the enzyme to the corresponding 2-hydroxycinnamic acids. Other classes of CAs among the eight currently known do not possess esterase activity or this activity was poorly investigated. Hence, we decided to look at the potential of coumarins as inhibitors of the η-CA from the malaria-producing protozoan *Plasmodium falciparum*, PfaCA. A panel of simple coumarins incorporating hydroxyl, amino, ketone or carboxylic acid ester moieties in various positions of the ring system acted as low to medium micromolar PfaCA inhibitors, whereas their affinities for the cytosolic off-target human isoforms hCA I and II were in a much higher range. Thus, we confirm that η-CAs possess esterase activity and that coumarins effectively inhibit this enzyme. Elaboration of the simple coumarin scaffolds investigated here may probably lead to more effective PfaCA inhibitors.

## 1. Introduction

Comarins were discovered relatively recently to act as inhibitors of the zinc enzyme carbonic anhydrase (CA, EC 4.2.1.1)^1^. Unlike all other inhibitor classes investigated at that time, surprisingly, these compounds were shown to not coordinate to the metal ion from the α-CA active sites (the human isoforms hCA I – XIV were initially investigated for their interaction with these compounds^1^^,^^2^) but to bind at the entrance of the active site cavity. In addition, the coumarin lactone ring was found hydrolysed to the corresponding 2-hydroxycinnamic acids (either in *cis*- or *trans* geometry) making these compounds the first reported class of pro-drug CA inhibitors (CAIs). Thus, a rather large number of drug design studies were performed over the last decade^2–4^ using both natural products as well as synthetic coumarins as starting point, which established the fact that coumarjns are among the most effective and isoform-selective CAIs known to date^1–4^. Indeed, derivatives with selectivity for all human isoforms have been reported so far, although the largest number of studies and derivatives investigated to date were designed for targeting the transmembrane, cancer-associated isoforms hCA IX and XII, which are validated antitumor/antimetastatic drug targets^5^^,^^6^.

However, up until now, coumarins were not investigated for their interactions with non-α-CAs. In fact, among the eight reported CA genetic families (the α – ι-CA classes^7^^,^^8^) known so far, only the α-CAs were investigated in detail for their catalytic versatility, and they possess indeed a rather effective esterase as well as other catalytic hydratase/hydrolase activities^9^. Generally, other CA classes than the α-family do not possess esterase activity, although there are several erroneous reports of such an activity for β- and δ-CA enzymes^10^, which have been shown by other groups to be artefactual data^11^. However, the η-CAs, present in protozoans belonging to the genus Plasmodium, PfaCA^12^, which have originally been annotated as being α-CAs, are known to possess esterase activity with 4-nitrophenyl acetate as substrate^13^. They were subsequently shown to represent a new CA family, the η-class, and also proposed as a potential anti-malarial drug target^12^^,^^14^. However, apart the initial reports from Krungkrai’s group^13^, which undoubtedly showed that PfaCA has esterase activity with 4-nitrophenyl acetate as substrate, and that this activity is potently inhibited by primary sulphonamides^13^, the main class of zinc-binding CAIs^15^, no detailed such studies on this enzyme were performed. It should be stressed that after we showed that PfaCA is not an α- but an η-CA^12^, a multitude of sulphonamide and anion inhibitors of this enzyme (both for a truncated as well as for a longer form of it) have been detected, some with potency in the low nanomolar range (for the sulphonamides and their derivatives)^16^.

Here we show that coumarins indeed act as PfaCA inhibitors, which is only possible due to the esterase activity of PfaCA, the prototypical η-class CA. In a small series of simple such derivatives, inhibition constants in the micromolar range against PfaCA were detected, and, more interestingly, many of the investigated coumarins were more effective protozoan enzyme inhibitors compared to their activity on the off-target human isoforms hCA I and II.

## 2. Materials and methods

### 2.1. Enzymology and CA activity and inhibition measurements

The CA-catalysed CO_2_ hydration activity has been measured with an Applied Photophysics stopped-flow instrument^17^. The used pH indicator was phenol red (at a concentration of 0.2 mM), working at the absorbance maximum of 557 nm. 10 mM HEPES (pH 7.4) was employed as a buffer, in the presence of 10 mM NaClO_4_ to maintain the ionic strength constant. The initial rates of the CA-catalysed CO_2_ hydration reaction were followed up for a period of 10–100 s. The substrate CO_2_ concentrations ranged from 1.7 to 17 mM for determining the inhibition constants. For each inhibitor, at least six traces of the initial 5–10% of the reaction were used to determine the initial velocity. The uncatalyzed rates were determined in the same manner and subtracted from the total observed rates. Stock solutions of inhibitors (10 mM) were prepared in distilled-deionized water with maximum 5% DMSO, and dilutions up to 10 nM were done thereafter with the assay buffer. Inhibitor and enzyme solutions were preincubated together for 1–6 h prior to the assay, in order to allow for the formation of the E-I complex. The inhibition constants were obtained by non-linear least-squares methods using Prism 3 and the Cheng-Prusoff equation, as reported previously^18–20^, and represent the mean from at least three different determinations. The PfaCA concentration in the assay system was 12.38 nM. The human/protozoan enzymes were recombinant proteins obtained in-house, as described earlier^12^^,^^16^.

### 2.2. Chemistry

Coumarins **1–14**, buffers, acetazolamide **AAZ** and other reagents were of >99% purity and were commercially available from Sigma-Aldrich (Milan, Italy).

## 3. Results and discussion

As mentioned in the introductory part, Krungkrai’s group first report that the *Plasmodium falciparum* genome encodes for CAs, which have been assigned to the α-class^13^. In these initial studies, the esterase activity of such an enzyme, later denominated PfaCA^12^ has been observed, working with 4-nitrophenyl acetate as substrate, and indeed, the enzyme showed a significant such activity, which has been potently inhibited by primary sulphonamides and their isosteres^12^^,^^14^^,^^16^, that are among the most investigated classes of CAIs^15^.

**Figure 1.**
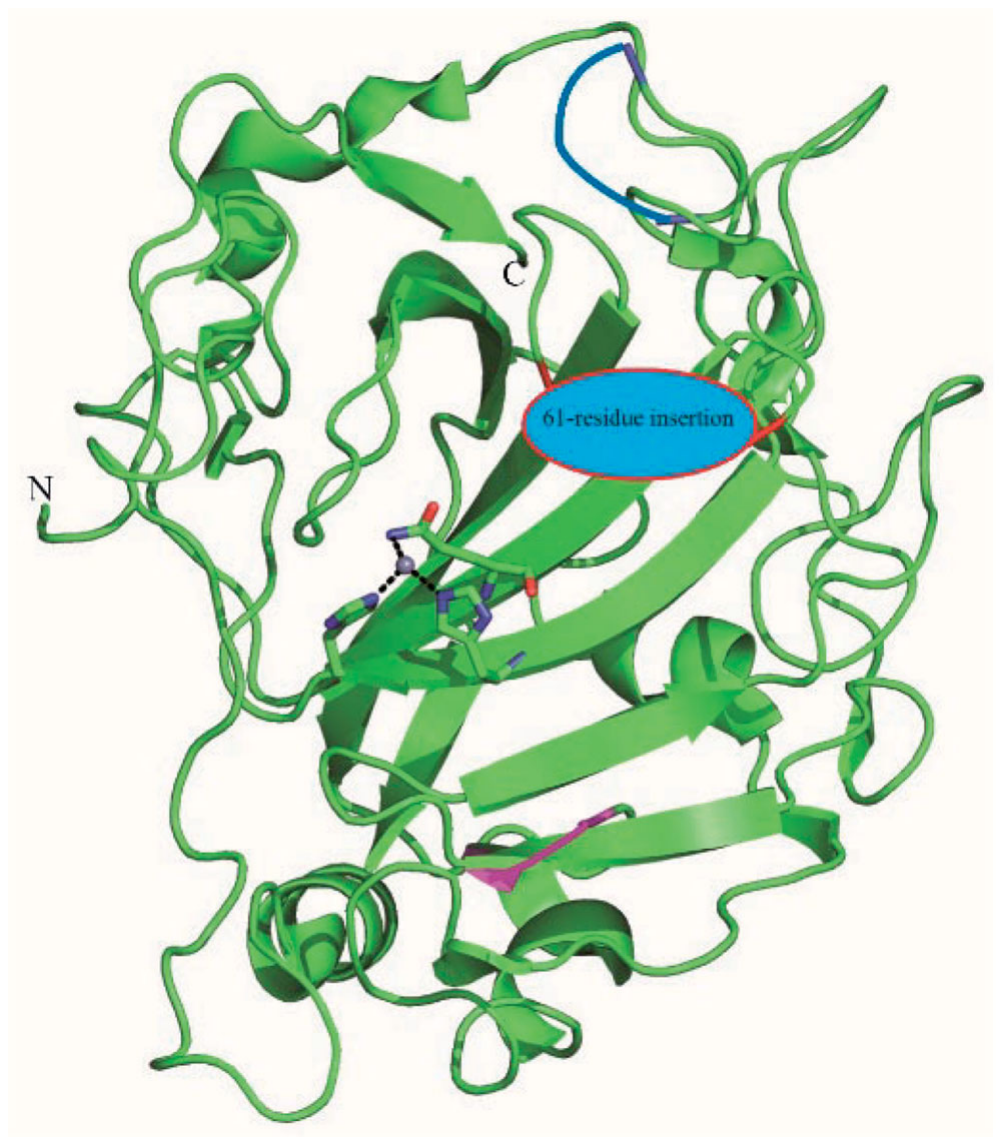
Homology modelling and coordination of the zinc ion in the active site of PfaCA. The zinc ion (central grey sphere) is coordinated by the imidazole moieties of residues His299, His301 and the nitrogen from the CONH_2_ moiety of Gln320^12^. The numbering of the amino acid residues is not shown for the sake of simplicity, but the 61 amino acid residues insertion which could not be modelled is highlighted in blue. The protein backbone is shown in green.

A closer look at the amino acid sequence of PfaCA and orthologs from other Plasmodium species, allowed us to observe that these enzymes do not possess the three His ligands that coordinate the Zn(II) ion in all α-CAs^21^, but instead the metal ion (which is crucial for catalysis) was proposed to be coordinated by two His and one Gln residues^12^. Indeed, a homology modelling study allowed us to propose the partial structure of the enzyme^12^, which could not be modelled entirely as the enzyme used was a truncated form, but part of the active site and especially the metal ion and its ligands could be clearly modelled and are shown in Figure 1.

Although no X-ray crystallographic data were obtained so far for PfaCA, a previous study from Christianson’s group showed that mutating the His zinc ligands from the human isoform hCA II, such as for example the His119Gln substitution, leads to an enzyme that has the zinc coordination pattern presented in Figure 1 for PfaCA, and this enzyme also preserves its catalytic activity for the CO_2_ hydration reaction^22^.

Such data prompted us to investigate the possible inhibitory activity of coumarins against PfaCA, which as mentioned above, must be hydrolysed by the esterase activity of the enzyme in order to generate the active inhibitor^1^.

The simple mono- and di-substituted coumarins incorporating hydroxyl, amino, ketone or carboxylic acid ester moieties in various positions of the ring system of types **1–14** included in this study are shown in Table 1, together with their inhibitory data against PfaCA and two off-target human isoforms, hCA I and II. The following structure-activity relationship (SAR) can be observed from the above data:

**Table 1.** Inhibition data of hCA I and II and protozoan enzyme PfaCA with coumarins **1–14** and acetazolamide (**AAZ)** as standard drug by a stopped-flow CO_2_ hydrase assay^17^.

Name	Structure	K_i_ (µM)^a^		
		hCA I	hCA II	PfaCA
**1** ^c^	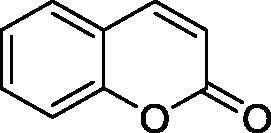	160.0 (3.1)^b^	600.0 (9.2)^b^	69.4
**2** ^c^	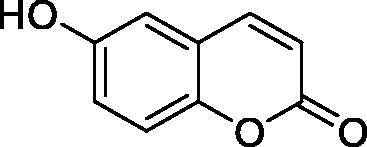	192.0	683.0	17.3
**3** ^c^	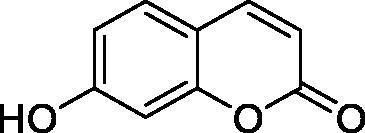	263.5	690.6	20.4
**4** ^c^	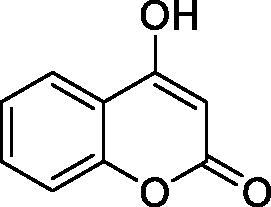	393.5	513.1	74.4
**5** ^c^	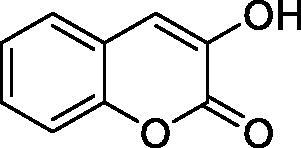	489.8	625.2	90.5
**6** ^c^	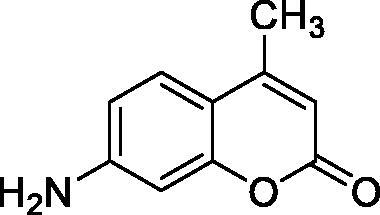	646.3	485.7	27.6
**7** ^c^	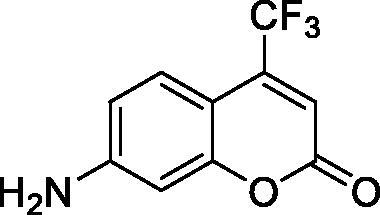	939.6	733.5	56.3
**8** ^c^	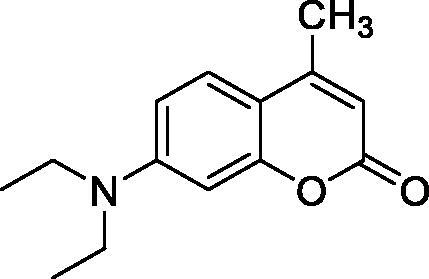	516.5	558.9	35.8
**9** ^c^	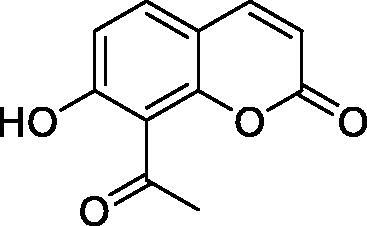	948.9	646.2	25.5
**10** ^c^	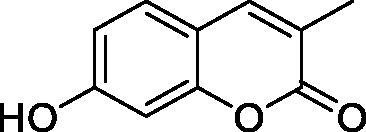	137.0	296.5	46.9
**11** ^c^	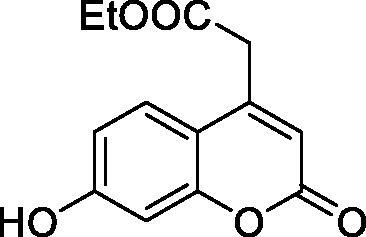	748.9	875.6	455.0
**12** ^c^	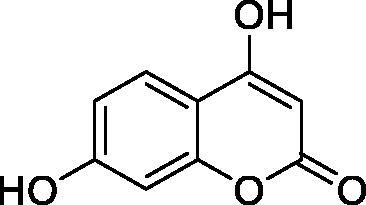	181.8	758.4	54.8
**13** ^c^	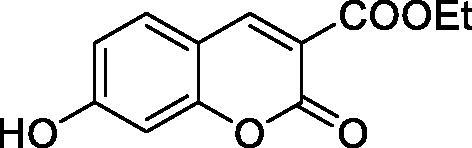	900.1	961.2	311.0
**14** ^c^	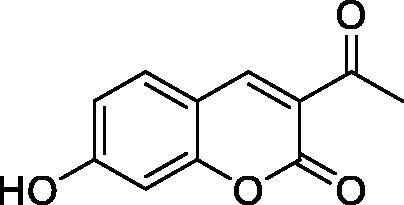	469.7	786.2	334.3
**AAZ**	–	0.25	0.012	0.17

^a^Mean from 3 different assays, by a stopped flow technique (errors were in the range of ± 5–10% of the reported values).

^b^Data from ref.^1^, using a different incubation time.

^c^Incubation time of 6 h.

- the most effective PfaCA inhibitors in the investigated series were **2, 3, 6, 8** and **9**, which showed inhibition constants ranging between 17.3 and 35.8 µM. The presence of OH moieties in positions 6- or 7- of the coumarin ring led to the most effective inhibitors (**2** and **3**, K_I_s of 17.3 – 20.4 µM), whereas amino, diethylamino, or methylketone groups (present in compounds **6, 8** and **9**) led to slightly less effective PfaCA inhibitors. The presence of substituents in position 4 of the coumarin ring led to a decrease of potency for the methyl-containing such derivatives (**6** and **8**), which was even more accentuated for the when CF_3_ (derivative **7**) or ethoxycarbonylmethyl (derivative **11**) groups were present.
- Medium potency PfaCA inhibition was observed with the following coumarins investigated here: **1, 4, 5, 7, 10** and **12**, which had K_I_s of 46.9 – 90.5 µM (Table 1). The unsubstituted coumarin 1 is thus a medium potency-weak inhibitor (K_I_ of 69.4 µM) but minor structural changes, such as the introduction of an OH group in positions 6 or 7, as shown above, drastically increase the inhibitory potency (derivatives **2** and **3** discussed above). However, the isomers with the OH group in positions 3 and 4 (compounds 4 and 5) showed a decrease of the inhibitory properties against PfaCA (K_I_s of 74.4 – 90.5 µM), demonstrating that these positions should be not substituted even with compact groups in order to obtain effective inhibitors. The same is true when a methyl is present in position 2, with compound **10** being 2.3 times a less effective PfaCA inhibitor compared to the de-methylated analog **3**.
- The least effective PfaCA inhibitors were **11, 13** and **14**, which showed K_I_s of 311.0–455.0 µM (Table 1). These compounds incorporate bulkier moieties in positions 2 or 3 of the coumarin ring compared to the other derivatives included in the study, clearly demonstrating that the best activity is probably obtained when the lactone ring is unsubstituted. Modifications leading to effective inhibitors should thus consider substitution patterns in positions 6, 7 and 8 of the coumarin. This situation was in fact observed also for the inhibition of the human CA isoforms hCA I-XIV already in the first studies in which coumarins were reported as CAIs^1^^,^^2^.
- The investigated coumarins were rather ineffective inhibitors of the human isoforms hCA I and II; with K_I_s in the range of 137.0–948.9 µM against hCA I and of K_I_s of 296.5–961.2 µM against hCA II. This is a relevant observation, as it demonstrates that the parasite enzyme is more inhibited than the human CAs included in the study.

## 4. Conclusions

This is the first study in which the inhibitory effects of coumarins against a non-α-CA are demonstrated. In a small series of mono- and di-substituted coumarins incorporating various substituents (OH, amino, Me, CF3, ketone, ethyl ester, etc.) and diverse substitution patterns, we demonstrate micromolar inhibition against PfaCA, a pathogen enzyme from the malaria provoking parasite *P. falciparum*. The SAR for obtaining effective PfaCA inhibitors is rather obvious, with the most effective compound having no substituents on the lactone ring and OH, amine or ketone groups in positions 6, 7 or 8 of the second ring. The study thus confirms that η-CAs possess esterase activity and that coumarins effectively inhibit this enzyme. Elaboration of the simple coumarin scaffolds investigated here may probably lead to more effective, presumably nanomolar PfaCA inhibitors, which might constitute interesting anti-malarial drug candidates.
